# Determination of Quality Changes in Peaches Wrapped in Active Paper and Stored at Ambient Temperature in Summer

**DOI:** 10.1038/s41598-017-09221-1

**Published:** 2017-09-19

**Authors:** Xiao-long Du, Hui Li, Wei-hong Zhou, Ying Liu, Jian-long Li

**Affiliations:** 1 Department of Ecology, School of Life Sciences, Nanjing University, Nanjing, 210046 China; 2 State Key Laboratory of Pharmaceutical Biotechnology and Collaborative Innovation Center of Chemistry for Life Sciences, School of Life Sciences, Nanjing University, Nanjing, 210023 China; 3 Suzhou Institute of Technology, Jiangsu University of Science and Technology, Zhangjiagang, 215600 China

## Abstract

Peaches are known for their palatable flavor and abundant nutrients. However, peaches are perishable, and the existing preservation techniques for peaches are still immature. To further extend the shelf life and prevent nutrient loss of perishable peaches under ambient temperature in summer (approximately 25–32 °C), we conducted experiments wrapping peaches (*Prunus persica* cv ‘Baihua’) in single- and composite-treated vegetal fibrous papers that contained calcium carbonate, phytic acid, Na-alginate and vitamin C. The pathogenic fungi that primarily caused peach decay during storage belonged to the genera of *Penicillium*, *Botrytis*, *Aspergillus*, *Alternaria*, and *Rhizopus*. After analyzing quality attributes, including weight loss, firmness, soluble sugar content, respiration rate, relative electric conductivity, malonaldehyde content, peroxidase activity and the decay index, we proved that vitamin C within the preservative paper greatly contributes to peach preservation. Combined with phytic acid and Na-alginate, the composite vitamin C preservative papers played significant roles in delaying fruit senescence, and 0.4% (w/v) vitamin C preservative paper with 1% Na-alginate could maintain quality and extend shelf life with the best effect. This preservation technique significantly postponed the respiration peak by 2–3 days and is a significant contribution to contemporary commercial production.

## Introduction

Peach (*Prunus persica*) is an important economic crop that is widely domesticated and cultivated worldwide with a long history^1^. Peaches are known for their palatable flavor and abundant nutrients, which include various types of amino acids, vitamins, phytochemicals and other nutrients. However, as honey peaches are generally harvested in areas with high temperature and high humidity for the entire summer and early autumn with a short after-ripening period, they experience rapid deterioration, which causes tremendous economic losses. Regardless of mechanical injuries, diseases and insect pest damage, the shelf life of a peach is only approximately 2–3 days. Peaches are classified as atypical respiration climacteric fruit that is severely restricted by internal physiological properties and external environments. The traditional preservation of peaches is cold storage with modified atmosphere packaging, which has drawbacks including increasing cost of facility purchase, energy consumption and frost injuries. Thus, it is essential and urgent to develop cheap, safe and efficient integrated preservation technology in fruits and vegetables under ambient temperature in summer (approximately 25–32 °C).

Currently, preservative paper has been widely applied for fruit preservation, and the production of vegetal fiber paper is more environmental friendly and cheaper than plastic film, which degrades slowly^2^. Additionally, using vegetal fibrous paper with preservatives can increase the preservation of fruits. In previous experiments, many preservation materials with vegetal fibrous paper have been tested by us and a few of them are used as experimental preservatives in this study. Calcium carbonate has physical properties of water absorption and densification and is used as an auxiliary for preservation products. Recently, calcium carbonate was added to Rich Mineral Paper (RMP, 70–80% calcium carbonate), making it a viable alternative to traditional fiber paper^3^. Calcium ions can inhibit hydrolase activity within cells. They stabilize the cell membrane structure and interact with the regulatory proteins on the membrane and calmodulin to induce a series of biochemical reactions within the cell that ultimately increase the shelf life of peaches^4^. Phytic acid is a native compound extracted from food crops. It has very strong metal chelating abilities and is an antioxidant, so it is widely applied in the food industry as a stabilizer, color fixative and preservative. Some studies have found that phytic acid effectively inhibits the decay and browning of apple fruit or juice as an enzyme inhibitor^5^. As a natural polysaccharide, sodium alginate (Na-alginate), which is extracted from brown seaweed and synthesized in microorganisms^6^, has features of nontoxicity, biodegradability, biocompatibility, reproducibility and low cost. It can easily form gels and films with divalent cations^7,8^. Liu^9^ found that applying 2% Na-alginate on fresh mango can effectively delay the respiratory peak. Zhu^10^ used Na-alginate composited film to prolong the shelf life of jujube fruit. Ascorbic acid, also known as vitamin C, also significantly slows down the decay of soluble sugar content and accumulation of lipid peroxidation products to maintain the integrity of the membranous antioxidant. Thus, vitamin C can effectively prevent enzymatic browning of fruits^11^ and flowers^12^.

Based on our rich experience in developing and leveraging green agricultural preservation products, the objective of this study was to estimate the preservation effect of specified preservatives with vegetal fiber paper on peaches over 6 days (d) of storage at ambient temperature (25–32 °C) in summer. Additionally, this research investigates optimal safe and effective integrated preservation techniques that can be applied in agricultural production. Many preliminary experiments have been conducted to determine the appropriate dosage of related reagents. Preservation effects are related to the following 8 quality attributes: weight loss, firmness, soluble sugar content, respiration rate, relative electric conductivity, malonaldehyde content, peroxidase activity and decay index. In addition, microbiological data were also obtained to further verify the preservation effect. The experimental peaches were harvested uniform in growth state, weight and shape. Preservative efficacy was tested for both single- and composite-treated groups.

## Experimental Designs

The experiments were performed in two sections. The first section was carried out to measure the effects of single preservation materials. The selected peaches were packaged with different types of preservative paper treated with calcium carbonate, phytic acid, Na-alginate or vitamin C for 6 d. The untreated group and another group only covered with vegetal fibrous paper were defined as the control check (CK) groups. In the second section, peaches were packaged with vitamin C paper treated with different concentrations of Na-alginate and phytic acid. Samples were collected and analyzed at a specific time point every day. Each treatment was repeated 3 times and averaged.

### The First Section - Treatment of Preservatives on Vegetal Fibrous Paper

The appropriate concentrations of preservatives were determined by preliminary experiments^13^. As is shown in Table 1, the single-treated preservative paper was prepared by immersing the vegetal fibrous paper in phytic acid (paper-2), Na-alginate (paper-3) or vitamin C (paper-4) at room temperature for 10 min. All samples were air-dried for approximately 30 min for dehydration. In addition, the preservative paper treated with calcium carbonate was replaced by the Rich Mineral Paper (paper-1), and the untreated bare peach was defined as CK.

**Table 1:** Composition of impregnation solutions used for different papers.

Name of Group	Treatments	
	Composition of Impregnation Solution (w/v)	Additional Remarks
Control check (CK)	N/A^a^	Untreated peach without package
Paper-0	N/A	Vegetal fibrous paper
Paper-1	Rich Mineral Paper	70–80% calcium carbonate (w/w)
Paper-2	Phytic acid (0.1%)	
Paper-3	Na-alginate (1%)	
Paper-4	Vitamin-C (0.4%)	
Paper-5	Phytic acid(0.05%), Vitamin-C (0.4%)	Impregnation solutions were mixed with adhesive of sodium salt (1%) and oxidized starch (16%)
Paper-6	Phytic acid(0.1%),Vitamin-C (0.4%)	
Paper-7	Phytic acid(0.15%),Vitamin-C (0.4%)	
Paper-8	Na-alginate (0.5%), Vitamin-C (0.4%)	
Paper-9	Na-alginate (1%), Vitamin-C (0.4%)	
Paper-10	Na-alginate (0.5%), Vitamin-C (0.4%)	

^a^N/A: not applicable.

### The Second Section - Treatment of Preservatives on Vitamin C Paper

Based on the results of the previous experiments, we designed composite Vitamin C preservative paper with phytic acid and Na-alginate as additives. The paper was made from 160 g soluble starch, 1.2 g potassium permanganate and 10 g sodium hydroxide dissolved in 1 L distilled water heated to 90–95 °C and stirred for 20–30 min to obtain the oxidized starch for adhesion of impregnation solutions. The product of sodium hydroxide and potassium permanganate after an oxidation reaction was sodium salt and insoluble manganese dioxide, which was filtered out for non-toxic. The composite vitamin C (0.4%, w/v) preservative papers were obtained by coating and air-drying with the appropriate preservative dissolved in the adhesion agent. Six treatments with different concentrations of phytic acid and Na-alginate on vitamin C preservative paper were established as paper-5 to paper-10 in Table 1. The original vitamin-C paper (Paper-4) was defined as the control group in this experiment. All experiments were conducted at ambient temperature and then placed in sterile plastic boxes to maintain moisture at 75–85%.

## Results

### Variations of Weight loss

**Figure 1:**
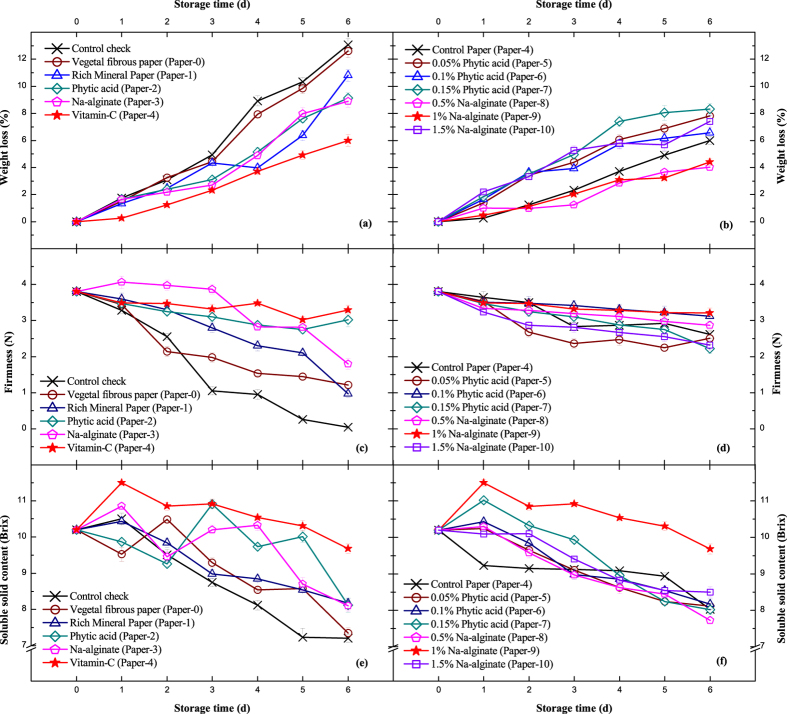
Weight loss (%), firmness (N) and soluble solid content (Brix) with standard deviation shown in error bars. (**a**), (**c**) and (**e**) Single treatments of vegetal fibrous paper. (**b**), (**d**) and (**f**) Composite treatments of vitamin C (0.4%) paper. Detailed information on the papers is provided in Table 1.

The postharvest weight loss of fruits is mainly caused by water loss and nutrition consumption in transpiration and respiration^14^. After 6 d of storage, the weight reduction of all groups gradually increased, and the weight loss ratio of the CK group was 12.17% greater than other packaged groups (Fig. 1a). The vitamin C treatment, with the lowest weight-loss ratio of 6.00%, was considered to have the optimal effect among the experimental treatments. Compared with untreated bare peaches, the external packing of vegetal fibrous paper could form a physical barrier to reduce the evaporation of water and block air exchange to inhibit respiration and nutrient consumption.

Based on single-treatment experiments, the composite vitamin C preservative papers with phytic acid and Na-alginate as additives were established in this study (Fig. 1b). Overall, the final weight losses of composite treatments were lower than those of single treatments. The quantitative values were well distributed and not significantly different. The vitamin C and vitamin C compounded with Na-alginate (Paper-9 and Paper-10) had a better effect on preservation at certain optimal concentrations, and phytic acid had no positive effect on maintaining weight combined with vitamin C paper. In our previous study, the coating of 2% pullulan (w/v) with 0.25 kJ/m^−2^ UV-C (ultraviolet-C rays) could reduce the weight loss of ‘Baihua’ peach to 15% after 7 d of storage^13^, which is not as good as the vitamin C preservative papers. Gao H. *et al*.^15^ found that 0.1 mmol/L melatonin treatment on the surface could delay postharvest senescence and reduce weight loss to 5–12%, and the ratio is largely affected by various characteristics and the external environment^16^.

### Firmness changes

Firmness is an important physical indicator for fruits in production and transportation. It is mainly influenced by status of endogenous pectin. Green fruits contain more protopectin than water-soluble pectin, which gradually dissolves in the natural maturity process^17^. The structure of cellulose, hemicellulose, pectin and extensin change, degrading the cell wall and directly causing fruit softening^18^. The result of Fig. 1c demonstrated that the physical protection of vegetal fibrous paper is especially necessary. The firmness of bare peaches decreased to 0.4 N, much lower than packaged ones after storage for 6 d. As shown in Fig. 1d, phytic acid, Na-alginate and vitamin C could efficiently enhance preservation. The firmness results in phytic acid, Na-alginate and composite vitamin C-treated groups (3 N on average) were generally higher than those in single-treated groups, among which the 0.1% phytic acid and 1% Na-alginate treatments had the best effect at 3.12 N and 3.21 N. The firmness of the experimental cultivar ‘Baihua’ is relatively lower than normal peach species, which have firmness levels of 8–40 N^13,19,20^, and this cultivar is more vulnerable to mechanical damage during transportation.

### Soluble Solid Content Variation

The soluble solid content refers to the total dissolved compounds, including sugars of soluble metabolites that mainly include glucose, fructose and sucrose. The soluble solid content gradually accumulates as a respiratory substrate to release energy until the peach is fully matured. The continual consumption of nutrients leads to decreased quality and shortens the postharvest shelf life of the peach^21^. From the results in Fig. 1e, the external packing in vegetal fibrous paper has minimal effect on inhibiting the loss of soluble solid content. The final value was the same as the CK group, and both values stayed at low levels of 7.0–7.5 Brix. Combined with the results from Fig. 1f, the first few days of the experiment were confirmed as an important post-ripening period for soluble solid content accumulation with regular increase because the experimental peaches are not fully ripened. The application of 1% Na-alginate best maintained the peach nutrients in both experiments. The nutrient value of the Paper-4 and Paper-9 treatments (9.5–10.0 Brix) was significantly higher than others at each time point. As the test peach spices ‘Baihua’ is a mid-late maturing honey peach cultivar, the sucrose content is relatively lower than early-maturing cultivars, which have maximum values of 16.0–45.0 Brix^22^. Furthermore, sugars in fruits significantly enhance the nutrient content and sweetness. This results in decreasing osmotic pressure of cells that give rise to fruit decay due to microorganism breeding^14^. The aromatic substances composed by soluble sugars could ultimately form glycosides, phytochemicals (pectin) and anthocyanin derivatives^23^. That is, changes in soluble solids content are also regulated by upstream genes and signal pathways.

### Respiration Intensity

**Figure 2:**
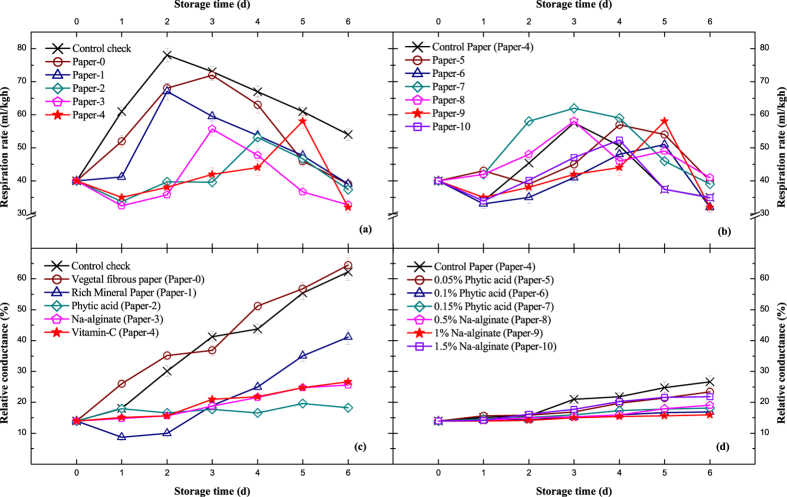
Respiration rate (ml/kgh) and relative conductance (%) with standard deviation shown in error bars. (**a**) and (**c**) Single treatments of vegetal fibrous paper. (**b**) and (**d**) Composite treatments of vitamin C (0.4%) paper. Detailed information on the papers is provided in Table 1.

Respiration intensity is an indicator of the strength of fruit metabolism. As the results show in Fig. 2a, peach is a typical fruit that exhibits climacteric respiration. All the packaged peaches showed a lower respiration ratio than bare ones. It is hypothesized that the sealed package of vegetal fibrous paper leads to an oxygen deficiency that inhibits aerobic respiration. In addition, accumulated carbon dioxide would also later inhibit anaerobic respiration. In general, the application of vitamin C on vegetal fibrous paper could effectively delay the arrival of respiratory peak by 3 days starting at approximately day 5. Combined with other additives as shown in Fig. 2b, the composite treated papers were confirmed to play good roles in suppressing fruit senescence by comparing the accumulated respiration. In general, the intensity of respiration is associated with other indicators, such as decreased firmness of flesh, increased soluble solids and relative electric conductivity, which is not good for fruit preservation^24^. As the experimental peach is more perishable than other varieties, the corresponding respiration rate (30–80 mL/kgh) is higher than common species (12–35 mL/kgh)^25^. Extensive experiments have concluded that the appearance of the respiration peak of fruit ripening is closely related to ethylene^26^. The respiration rate increased with the addition of exogenous ethylene and immediately decreased as ethylene was removed in hormone stress experiments, and that process is reversible. To further reduce the respiration influenced by ethylene, artificial regulation at the gene expression level is being performed simultaneously.

### Relative Electric Conductivity Change

In the process of fruit senescence, cell apoptosis and nutrient degradation seriously affect the normal function of the cell membrane, which becomes more permeable^27^. The released electrolytes are the main explanation for the relative electric conductivity change. From the results in Fig. 2c, the inhibition of moisture loss and respiration rate does not seem to prevent fruit senescence, as the variation tendency of the vegetal fibrous paper group is the same as the CK, and both of them approached a high level of 60–70%. In addition, the antistaling agents remarkably held the value of other groups to below 30%, except for the rich mineral paper group, which was only reduced to 41.1%. As shown in Fig. 2d, the composite vitamin C paper decreased the values of relative electric conductivity to below 30%. This was a marked advance over the single-preservative method and can be recognized as an effective method. The variation speed of relative electric conductivity in the later period is relatively faster, and the values of badly rotted peaches are extremely high because the decomposition of cells and pathogen breeding has started. Some studies indicated that electric relative conductivity has a significant correlation with fruit firmness, and it is also related to water content in plant tissues^13^. It is usually considered in the study of plant drought resistance, cold resistance, disease resistance and so on^28–30^. Therefore, keeping a low level of relative electric conductivity is very important to maintain the normal function of the cells.

### Malonaldehyde Content Variation

In the later period of fruit senescence, the cytomembrane of mesocarp cells begins to degrade, and malonaldehyde (MDA) is atypical final decomposition product of membrane lipid peroxidation. The MDA level directly reflects the degree of peach injury. The release of MDA from the membrane can also react with proteins and nucleic acids to unfasten the bonds between the cellulose molecules. Therefore, the accumulation of MDA is severely harmful to the membrane and cells^31^.

**Figure 3:**
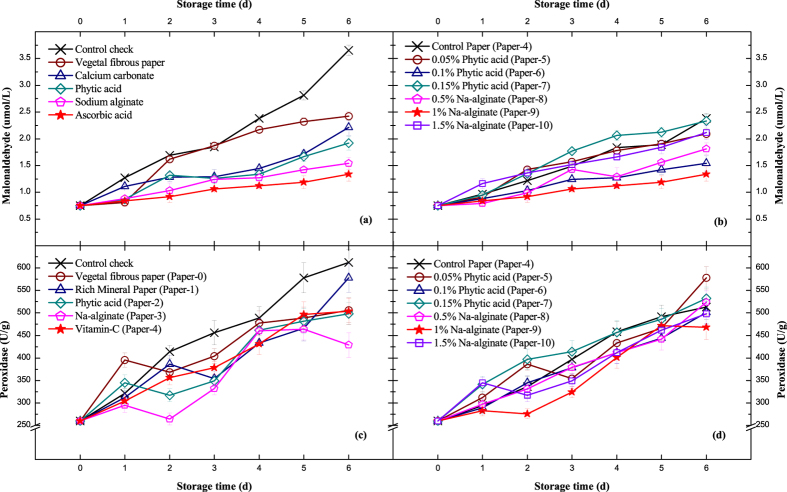
Malonaldehyde (μmol/L) and peroxidase (U/g) with standard deviation shown in error bars. (**a**) and (**c**) Single treatments of vegetal fibrous paper. (**b**) and (**d**) Composite treatments of vitamin C (0.4%) paper. Detailed information on the papers is provided in Table 1.

We noticed the antistaling agents adhered on the paper, not the vegetal fibrous paper itself, effectively prevent an increase in relative electric conductivity, but the slowly increase of MDA values shown in Fig. 3a indicate that the variation speed of cell apoptosis is significantly faster than nutrient decomposition, and this may further result in invasions of various bacteria. In Fig. 3b, there are not many differences in the concentration of MDA among groups with composite treated additives, and the average value is slightly lower than the single-treatment ones. Overall, the 1% Na-alginate with 0.4% vitamin C group (Paper-9) displayed the best result of inhibiting the formation of MDA, which will help to extend the shelf life of peaches. Wang, J. *et al*.^32^ found that 10–20 kPa hypobaric treatment could reduce the MDA values of experimental peaches by 2–5%, which could also be applied in our experiments to enhance the preservation effect.

### Peroxidase Activity

Peroxidase (POD) catalyzes the oxidation of phenols through hydrogen peroxide to produce quinone compounds, which can further be condensed or combined with other molecules to form darker ubiquinone compounds^33^, and this is what is commonly called browning. The emergence of browning leads to decreases in fruit appearance and quality. It should be avoided as much as possible.

Figure 3c,d shows that the POD activity gradually increased to 450–600 U/g with aging of the peach in two categories of preservation treatments after 6 d of storage, and even the changing trends are similar. In comparison to Fig. 3c, the value distributions of the composite method in Fig. 3d are more compact, which is probably influenced by vitamin C. The specific details are still unclear and need further study. As an important indicator of biological enzyme in postharvest fruits, POD activity has been studied extensively. Huan, C. *et al*.^34^ demonstrated that treatment with heat and 1-MCP (1-methylcyclopropene) could delay the postharvest ripening process of peach fruit by regulating functional genes related to POD at the molecular level. POD is also considered an important indicator in research of plant stress tolerance, growth, genetics, breeding, lignin biosynthesis and other physiological processes^35,36^.

### Decay Degree

The decay index is calculated based on coverage condition of rotten spots on the fruit surface. A higher decay index indicates a higher degree of decay. According to our detailed sorting parameters, the peaches were confirmed to have good appearance and to be mostly nutrient at a decay index below 25%. If the decay index is 25–40%, the peach generally must be sold at a discount. When the decay index is over 40%, the peaches are unfit for eating, and this seriously affects the economic benefit. Therefore, the decay index is a referable economical index for peach industry.

The statistics for all treatments were collected every day during the experiments, and Table 2 showed the decay index (%) of single-treated groups over 6 d of storage. Obviously, the shelf life of peaches at room temperature of 25–32 °C were generally delayed by 1–2 days, and the vitamin C treatments had the best effect. Non-treated peaches (CK) became completely rotten and inedible (DI = 100%) after only 5 days. This result further confirmed the short shelf life of the experimental peach (*Prunus persica* cv ‘Baihua’) in the summer.

**Table 2:** The decay index (%) of single-treated groups over 6 d of storage.

Storage time(d)	Group Name					
	Control check	Vegetal fibrous paper (Paper-0)	Rich Mineral Paper (Paper-1)	0.1% Phytic acid (Paper-2)	1% Na-alginate (Paper-3)	0.4% Vitamin-C (Paper-4)
0	**0**	**0**	**0**	**0**	**0**	**0**
1	**11.5** ± **0.2**	**0**	**0**	**0**	**0**	**0**
2	**21.3** ± **0.8**	**14.6** ± **0.2**	**11.5** ± **0.2**	**0**	**0**	**0**
3	48.9 ± 0.8	38.5 ± 0.8	**23.9** ± **0.7**	**10.4** ± **0.4**	**8.4** ± **0.1**	**5.2** ± **0.1**
4	81.3 ± 2.3	63.5 ± 1.4	51.1 ± 2.2	29.1 ± 1.0	30.2 ± 1.1	**23.9** ± **0.4**
5	100	90.6 ± 3.1	69.8 ± 2.5	56.2 ± 2.1	60.4 ± 2.5	48.9 ± 1.8
6	100	96.9 ± 3.5	82.0 ± 3.7	73.9 ± 3.8	70.8 ± 2.7	62.5 ± 1.6

^*^Bold numeric values represent well-preserved peaches, underlined numeric values represent salable peaches and the remaining numeric values represent inedible peaches. Detailed information on the papers is provided in Table 1.

As the decay index (%) of composite-treated vitamin C (0.4%) papers shown in Table 3, the shelf life of peaches were efficiently delayed by 2–3 days over 6 d of storage, the 0.1% phytic acid (Paper-6) and 1% Na-alginate (Paper-9) treatments play the best effects. Combined with the results of single-treated groups, it is obvious that the development of decay on the surface of peaches is very fast for only few groups of peaches showed buffer values (25–40%, yellow block). Because the decay index is a quantitative index of appearance, the corresponding descriptions in different studies of postharvest fruits may vary. However, it could be a convenient reference for consumers in the market.

**Table 3:** The decay index (%) of composite-treated vitamin C (0.4%) papers during 6 d of storage.

Storage time(d)	Group Name						
	Control paper (Paper-4)	0.05% Phytic acid(Paper-5)	0.1% Phytic acid(Paper-6)	0.15% Phytic acid(Paper-7)	0.5% Na-alginate (Paper-8)	1.0% Na-alginate (Paper-9)	1.5% Na-alginate (Paper-10)
0	**0**	**0**	**0**	**0**	**0**	**0**	**0**
1	**11.5** ± **0.8**	**0**	**0**	**0**	**0**	**0**	**0**
2	**21.3** ± **0.9**	**0**	**0**	**0**	**0**	**0**	**0**
3	48.9 ± 1.4	**7.3** ± **0.2**	**0**	**4.2** ± **0.3**	**2.1** ± **0.1**	**0**	**3.1** ± **0.1**
4	81.3 ± 2.6	**25.0** ± **0.3**	**15.6** ± **0.4**	**22.9** ± **0.5**	**22.9** ± **0.8**	**17.7** ± **0.3**	**21.9** ± **0.3**
5	100	52.1 ± 1.2	38.5 ± 0.7	40.6 ± 1.8	42.7 ± 0.9	33.3 ± 0.9	41.7 ± 1.3
6	100	66.6 ± 3.2	57.3 ± 1.6	64.6 ± 1.6	58.3 ± 1.5	46.9 ± 1.1	65.6 ± 2.2

^*^Bold numeric values represent well-preserved peaches, underlined numeric values represent salable peaches and the remaining numeric values represent inedible peaches. Detailed information on the papers is provided in Table 1.

### Microbiological Data

After careful screening and culturing, we isolated and identified the pathogenic fungi that primarily caused peach decay during storage by microbial morphology and DNA sequence alignment. As shown in Table 4, they mainly included the genera of *Penicillium*, *Botrytis*, *Aspergillus*, *Alternaria*, and *Rhizopus*. In this study, the quantity of *Rhizopus*was relatively lower than that normally reported due to its strong pathogenicity in postharvest fruits^37^, and detailed information is still under investigation. The Colony-Forming Units (CFU) of these pathogenic fungi in experiments using composite vitamin C preservative paper with 0.1% phytic acid and 1% Na-alginate as additives was calculated by the plate count method after 6d storage. Packaging peaches in vegetal fibrous paper could reduce the proliferation of various pathogens to a certain extent, and it had greater effects in combination with specific compositions of vitamin C, phytic acid and Na-alginate. The composite vitamin C preservative papers reduced approximately 30–50% of multiplication number in total, which would be helpful to avoid the risk of food poisoning. Management technologies for postharvest disease are increasingly complex and expensive^38^, so the application of microbiological materials, such as antagonistic bacteria and antibiotic metabolites, will provide a wide space for intensive study of postharvest peach preservation.

**Table 4:** Levels (CFU/g) of identified pathogenic fungi in peach after 6 d of storage.

Category	Control check	Vegetal fibrous paper (Paper-0)		0.1% Phytic acid and 0.4% Vitamin-C (Paper-6)		1% Na-alginate and 0.4% Vitamin-C (Paper-9)	
	Levels (CFU/g)	Levels (CFU/g)	Decreasing rate (%)	Levels (CFU/g)	Decreasing rate (%)	Levels (CFU/g)	Decreasing rate (%)
*Penicillium* spp.	6.8 × 10^2^	5.4 × 10^2^	20.6	4.7 × 10^2^	30.9	4.5 × 10^2^	33.8
*Botrytis* spp.	5.0 × 10^2^	4.2 × 10^2^	16.0	2.8 × 10^2^	44.0	3.4 × 10^2^	32.0
*Aspergillus* spp.	7.5 × 10^2^	5.3 × 10^2^	29.3	3.6 × 10^2^	52.0	4.2 × 10^2^	44.0
*Alternaria* spp.	3.3 × 10^2^	2.6 × 10^2^	21.2	1.5 × 10^2^	54.5	1.7 × 10^2^	48.5
*Rhizopus* sp.	1.1 × 10^2^	0.9 × 10^2^	18.2	0.4 × 10^2^	63.6	<0.1 × 10^2^	>90.9

Note. sp. represents one species, and spp. represents two or more species. Detailed information on the papers is provided in Table 1.

## Discussion

We isolated the pathogenic fungi that primarily caused peach decay and identified them by microbial morphology and DNA sequence alignment. They belong mainly to the genera of *Penicillium*, *Botrytis*, *Aspergillus*, *Alternaria*, and *Rhizopus*. Their presence varied with the degree of peach decay.

By comparing the 8 quality attributes of weight loss, firmness, soluble sugar content, respiration rate, relative electric conductivity, MDA content, POD activity and decay index, the single-treatment experiments proved that the vitamin C preservative paper optimizes the preservation of peaches. In addition, we found that vegetal fibrous paper is cheaper and more environmentally friendly and can establish a sealing condition to improve the preservation of peach to a certain extent. In addition to vitamin C, phytic acid and Na-alginate both have good performances on enhancing the preservation effect.

Composite treatments experiments verify that composite antistaling agents adhered on vegetal fibrous paper outperform single-treated groups. The 0.4% (w/v) of vitamin C paper with 1% (w/v) of Na-alginate maximizes peach fruit quality and extends shelf life. For untreated peaches, it appears that the process of fruit senescence advanced rapidly in 2–3 days and then significantly slowed down. At a molecular level, the relative genes of affecting physico-chemical features through signal pathways require further in-depth studies.

In conclusion, this study presented the optimal effects of composite vitamin C preservative paper on different aspects of peach storage. We have proven that our techniques and procedures are easy to perform and meet food safety and green agriculture criteria. We believe our findings can be used to effectively enhance the preservation of postharvest peaches and add commercial value.

## Materials and Methods

### Fruit Material

Honey peaches (*Prunus persica* cv ‘Baihua’) were obtained in Fenghuang town, Zhangjiagang, China. Peaches at 70–80% maturity were harvested early in the morning as experimental materials. All of the selected fruits were approximately consistent sizes (7.74 cm on average diameter) and weights (216.7 g on average) with no mechanical damage or worms. They were immediately treated with experimental processing (day 0) in a constant environment of 23–25 °C and 60–65% relative humidity. Eight fruits were regarded as a replicate, and 3 replicates were tested per treatment.

### Weight Loss

The weight loss of fruit mass was measured by an electronic balance (±0.01 g, G&G Measurement Plant, Changshu, China). The weight loss ratio W (%) was calculated as follows:

1 $$W( \% )=\frac{{M}_{1}-{M}_{2}}{{M}_{1}}\times 100 \% $$

where *M*_1_ represents the initial weight of sample, and *M*_2_ represents the current weight of sample.

### Firmness

Firmness in different stages was evaluated by a sclerometer (DeFelsko, AT-100) equipped with a 0.8 cm diametric probe at a uniform velocity of 10 mm/min. Three measurements of peeled peach at different points of maximal axial diameter were obtained in each replicate, and the values were described as kilogram-force per square centimeter (kgf/cm^2^).

### Soluble Solids Content

Soluble sugar content was determined by refractometry (Shanghai Jinghua Science & Technology Instruments Co., Ltd., Shanghai, China) with anthrone reagent^39^. The peach flesh samples (1.0 g) were obtained and ground in test tubes that contained 25 mL of deionized water. The mixture was heated in a boiling water bath for 40 min and then cooled to ambient temperature. The contents of soluble sugar were calculated by absorbance at 630 nm.

### Respiration Rate

Eight hundred grams of peaches of different replicates were selected and placed in gas-tight containers at 2 °C. The containers were gradually flushed with scrubbed CO_2_ and humidified air at a flow rate of 20 mL/s. The real-time production rate of CO_2_ was measured in the flow-through system by gas chromatograph (Shimadzu GC-14, Kyoto, Japan) equipped with a thermal conductivity detector^40^. The respiration rate was expressed as mg CO_2_/kg/h.

### Relative Electric Conductivity

Relative electric conductivity was determined by conductivity meter (Model DDS-11A, Shanghai Scientific Instruments Co., Ltd., Shanghai, China). Peach slices of 10 mm in diameter and 4 mm thick were obtained and dried with filter paper in 50 mL conical flasks that contained 40 mL deionized water, then kept at 25 °C for 180 min and heated in a boiling water bath for 30 min. As the solution cooled to 25 °C, the final conductivity was calculated by the equation given below^41^.

2 $$\mathrm{REC}( \% )=({\rm{Relative}}\,{\rm{electrolyte}}\,{{\rm{leakage}}}_{{\rm{final}}}/{\rm{Total}}\,{\rm{electrolyte}}\,{\rm{leakage}})\times {\rm{100}}$$

### Malonaldehyde Content

MDA concentration was measured according to the procedures used by Dhindsa^42^. Sample tissues (2.0 g) were ground in liquid nitrogen with 5 mL of 10% (w/v) trichloroacetic acid (TCA). After centrifugation at 10,000 rpm for 15 min, 2 mL of the supernatant was taken and mixed with 2 mL of 10% (w/v) TCA containing 0.6% (w/v) thiobarbituric acid, then the mixture was heated to 100 °C for 20 min and quickly cooled before 10 min centrifugation at 10,000 rpm. The supernatant was collected and absorbance at 532, 600 and 450 nm was measured by spectrophotometer (Shanghai Jinghua Science & Technology Instruments Co., Ltd., Shanghai, China). MDA concentration (mol/L) was calculated employing the following equation:

3 $$MDA=6.45\times ({A}_{532}-{A}_{600})-0.56\times {A}_{450}$$

### Peroxidase Activity

POD activity was measured by following the method of Maehly^43^. Sample tissues (0.25 g) were ground with 0.01 g polyvinyl pyrrolidone and 5 mL 0.1 mol/L phosphate buffer (pH 7.8, 0.2 mmo1/L ethylene diamine tetraacetic acid and 0.4 mmo1/L β-mercaptoethanol). After centrifugation at 12,000 rpm for 10 min, the supernatant was mixed with guaiacol and then heated to dissolve the solutes and quickly cooled down; then, 28 µL 30% H_2_O_2_ was added for measurement. The POD activity was calculated as ΔA_470_/min/gFW.

### Decay Index

Disease severities of one peach in each group were assessed according to empirical scales as follows: level 0 represents a healthy peach; level 1 represents 1 to 3 lesions less than 3 mm in diameter; level 2 represents 25–50% surface damage of a peach; level 3 represents 50–75% surface damage of a peach; and level 4 represents more than 75% surface damage of a peach. The decay index was calculated by the formula below:

4 $$DI=\frac{\sum (df)}{ND}\times 100 \% $$

where d represents the degree of decay, f represents respective numbers, N represents the total number of examined peaches, and D represents the highest degree of disease severity^44^.

### Microbiological Test

The pulp and pericarp tissues at the junction of the decaying area (0–5 mm) of peaches were taken as testing materials. After isolation and cultivation in Plate Count Agar Medium (5.0 g tryptone, 2.5 g yeast extract, 1.0 g glucose and 15.0 g agar were boiled to dissolve in 1000 mL distilled water, the pH was adjusted to 7.0, and the mixture were sterilized at 121 °C for 20 min to prepare Plate Count Agar Medium) to form scattered colonies, the exogenous fungi that infected the peach were identified by 18S rDNA analysis. The DNA of different isolated fungi was extracted by DNAiso Reagent (TaKaRa). Subsequently, the 18S rDNA libraries were constructed by PCR (polymerase chain reaction), and the primer design was based on the conserved sequences of fungal 18S rDNA. The amplified genes were then purified and sent to Sangon Biotech (Shanghai, China)for sequencing and compared with the NCBI gene bank. The pathogenic fungi were identified by BLAST (https://blast.ncbi.nlm.nih.gov/Blast.cgi) from the American National Center for Biotechnology Information. The clones with 97% similarity of 18S rDNA with a known species were identified as that species. Afterwards, the antibacterial effects of different treatments on the paper were determined by detection of plate counts. The appropriate dilution multiples of sample tissues were cultured in Plate Count Agar Medium at 36 °C for 48 h to obtain the related statistics^45^. The final results were reported according to the distribution of colony counts (30–300) in 3 proper diluted concentrations.

### Data Processing

The experimental results were analyzed by Excel2007 and SPSS22.0 for Windows. Analysis of variance (ANOVA) and Duncan’s multiple ranges at a confidence interval of p = 0.05 were applied to determine the statistically significant differences among different treatments. The analysis graphics were created by Origin8.0.
